# Apple Extract as an Effective Silver Reducer in the Synthesis of Ag Nanoparticles

**DOI:** 10.3390/nano15080595

**Published:** 2025-04-12

**Authors:** Anna Wasilewska, Anna Basa, Monika Zambrzycka, Izabela Swiecicka, Beata Kalska-Szostko

**Affiliations:** 1 Doctoral School of University of Bialystok, Ciolkowskiego 1K, 15-245 Bialystok, Poland; 2 Faculty of Chemistry, University of Bialystok, Ciolkowskiego 1K, 15-245 Bialystok, Poland; 3 Faculty of Biology, University of Bialystok, Ciolkowskiego 1J, 15-245 Bialystok, Polandizabelas@uwb.edu.pl (I.S.); 4 Laboratory of Applied Microbiology, University of Bialystok, Ciolkowskiego 1J, 15-245 Bialystok, Poland

**Keywords:** silver nanoparticles, green synthesis, natural extracts, physicochemical characterization, antimicrobial properties

## Abstract

Nowadays, there is a growing need to develop environmentally friendly procedures that reduce the use of toxic chemicals in synthesis. Green synthesis methods have an advantage over conventional chemical methods because they do not pollute the environment significantly. This has generated more interest in using readily available plants to create nanomaterials. In this work, silver nanoparticles were obtained through green chemistry using natural reducing agents present in apple extract. The research focused on optimizing the synthesis conditions to obtain predictable structures. The characterization of the nanoparticles was performed using transmission electron microscopy (TEM), dynamic light scattering (DLS), X-ray diffraction (XRD), UV–Vis spectroscopy, and infrared spectroscopy (IR). The achieved results led to the conclusion that the use of apple extract was suitable for obtaining homogenous and spherical silver nanoparticles at a wide range of core precursor concentrations and a variable pH. The diameter of the studied nanoparticles ranged from 6 to 22 nm. The nanoparticles obtained with apple extract were highly active against Gram-positive bacteria and fungus, but less active against Gram-negative bacteria. The development of nanotechnology in green chemistry processes will gradually increase with technological advances, being a key component in developing new synthesis processes for nano-object formation.

## 1. Introduction

In recent years, the achievements in nanotechnology have been significant, with promising new methods to combat microorganisms, including pathogenic ones [1,2]. Some methods of synthesizing nanoparticles or nanocomposites have led to specific physicochemical and biological properties [3]. Therefore, researchers are particularly interested in nanoparticles produced by bioinspired methods. In particular, the synthesis of nanoparticles using plant extracts seems to be effective and profitable from both an environmental and economic point of view [4]. Therefore, such methods can be an economical alternative to large-scale production and an option for the recycling of organic waste [5,6]. Natural extracts are often effective stabilizers and diminish chemical consumption through compounds that have stabilizing and reducing properties [7,8]. Moreover, these compounds are also readily biodegradable [9,10]. Indeed, using aquatic environments and renewable raw materials, such as plant extracts, offers a wide range of possibilities for the further development of ecological methods of synthesizing metal nanoparticles [11,12].

In this study, we used apple extract as a reductant for the synthesis of silver nanoparticles. Apples contain (i) numerous vitamins, such as vitamins C, A, B, E, and K, tocopherols, and retinol; (ii) triterpenoids; (iii) anthocyanins; (iv) phenolic compounds; and (v) pectins [13,14,15]. Phenols, coumarins, ubiquinones, terpenoids, glycosides, alkaloids, and tannins may be responsible for the reduction of ions during the synthesis of nanoparticles [16]. In addition, the peptides and proteins present in apple extract, with a cysteine side chain, may also promote both ion reduction and the production of nanoparticles [17,18]. The carbonyl groups of proteins show a strong ability to bind metal nanoparticles, which means that proteins can form surface layers on these nanoparticles [19]. In turn, this prevents agglomeration and increases the stability of the synthesized objects in the aquatic environment [20]. Along with sugars, ethylene groups in apple extracts can also act as reducing agents, responsible for forming and aggregating the ions needed to create nanomaterials [20]. Due to the presence of reducing substances in plants (e.g., polysaccharides, plant cell wall components, and photosynthetic products) and various antioxidant compounds, the transformation of Ag^+^ to metallic Ag nanoparticles is possible [21,22,23,24]. In a reaction with plant extracts, silver nanoparticles may aggregate into larger particles, form clusters of biomolecules, or transform into various types of nanoparticles, such as AgCl [25]. The pH factor may also affect the size of nanoparticles [26]. Particles may destabilize at both an acidic and neutral pH, causing a faster aggregation rate. In an alkaline environment, nano-objects can exhibit stabilized super-structures due to the presence of hydroxyl ions [26].

Besides physicochemical characterization, another aspect of our research was understanding the antibacterial potential of nanoparticles synthetized with apple extract as a reducer. In general, there is a correlation between the bactericidal effect and the concentration of silver nanoparticles, e.g., bacterial cell growth resumed rapidly when the concentration of silver nanoparticles decreased [27].

A published study presented the effect of 12 nm silver nanoparticles on *Staphylococcus aureus* and *Escherichia coli*, using the broth microdilution method [28]. The results indicated that silver nanoparticles had no effect on *S. aureus* but showed high antibacterial activity against *Escherichia coli* at the minimal inhibitory concentration. This result was discussed as related to the difference in the structures of the cell walls of these bacteria (e.g., the presence of an outer membrane in Gram-negative bacteria such as *E. coli*) [28]. The antibacterial activity has been found to depend on the type of microorganism. Hence, we were motivated to test the antimicrobial activity of our nanoparticles against both Gram-positive (*Bacillus cereus*, *Staphylococcus aureus*) and Gram-negative (*Escherichia coli*) bacteria, as well as against a fungus (*Candida krusei*).

Although the synthesis of silver nanoparticles and their antimicrobial properties have been studied [29,30,31,32], our research provides a few novel aspects and focuses on a broader range of synthesis parameters. In one of the previous studies, silver nanoparticles were obtained using a single precursor concentration and a single sample [29], with an average particle size of 30 nm [29]. In our study, we aimed to investigate the effects of different silver nitrate concentrations on the sizes of the resulting nanoparticles, based on the hypothesis that lower precursor concentrations lead to the formation of smaller nanoparticles. Furthermore, in other studies [30], the synthesis process lasted up to 12 h, whereas our research demonstrates that nanoparticles can be obtained within a significantly shorter timeframe. Reducing the synthesis time not only increases the process’ efficiency but also enhances its alignment with the principles of green chemistry. Optimizing parameters such as the mixing time and temperature can further contribute to better control over the size and uniformity of the obtained nanoparticles.

Another approach [31] to silver nanoparticle synthesis involved the use of apple pulp, while, in our study, the entire fruit was subjected to thermal processing. This methodological approach may influence the chemical composition of the extract and its ability to reduce silver ions. Additionally, in recent studies [32], the antimicrobial potential of silver nanoparticles was assessed using the disk diffusion method. In contrast, our study employed the minimum inhibitory concentration (MIC) method, which provides more specific data on the antimicrobial activity of nanoparticles and allows for a more precise evaluation of the biological activity.

In summary, our research introduces significant innovations in the synthesis of silver nanoparticles by analyzing the effects of the silver nitrate concentration and pH, utilizing the whole fruit in the extraction process, and evaluating the antimicrobial properties through the MIC method. These aspects expand upon previous studies and contribute to advancements in the field of silver nanoparticles.

## 2. Materials and Methods

For the synthesis of the silver nanoparticles, we used silver nitrate (AgNO_3_), purchased from Sigma Aldrich (St. Louis, MO, USA), as well as acetone (C_3_H_6_O) and sodium hydroxide (NaOH), bought from AVANTOR (Avantor Performance Materials Poland S.A., Gliwice, Poland). We used AgNO_3_ as a silver ion precursor to synthetize nanoparticles of different sizes. The process of the purification and separation of nanoparticles from unreacted residues was carried out using acetone. An aqueous solution of a sodium base was applied to obtain nanoparticles in an alkaline medium, which were used to observe how the pH affected the synthesis of the nanostructures.

### 2.1. Synthesis of Silver Nanoparticles

Apples were bought from a local grocery store. Five different concentrations of silver nitrate were prepared (Table 1) to reduce the metallic particles and study the influence of the precursor concentration on the particles’ morphology, size, and composition.

- Apple extract preparation

Apples weighing 100 g were added to 200 mL of demineralized water; then, the solution was heated for 1 h at 80 °C. The obtained extract was filtered under a vacuum to remove apple residues. The pH of the obtained extract was equal to 2.1 [33].

- Synthesis of silver nanoparticles with apple extract

AgNO_3_ (Table 1) was used for the synthesis of nanoparticles at the concentrations of 0.25 mmol/L, 0.5 mmol/L, 1 mmol/L, 2 mmol/L, and 4 mmol/L. Distilled water was used as the solvent for the preparation of silver nitrate. The apple extract and the silver nitrate solution were combined in a 1:1 ratio. The solution was placed on a magnetic stirrer and heated at 80 °C for one hour [33]. After this, the solution was allowed to incubate for 24 h at room temperature. The next day, the product was centrifuged at 13,000 rpm for 30 min and dried in acetone.

- pH influence

To check the effect of the pH on the formation of nanoparticles, appropriate amount of 1 mol/L NaOH was added to samples B and C to obtain a more basic pH (Table 1). Prepared solutions B and C were divided into two parts in order to add the appropriate amount of sodium hydroxide. After adding the base, the solutions were heated at 80 °C for one hour.

**Figure 1:**
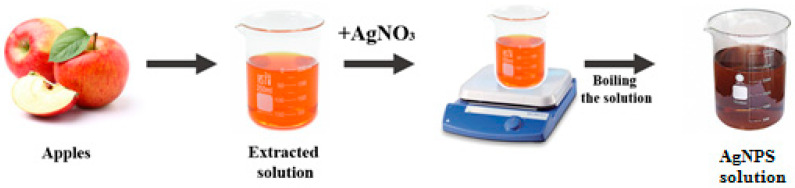
A schematic presentation of the steps followed to obtain silver nanoparticles based on apple extract.

Figure 1 shows a schematic presentation of the steps followed to achieve nanoparticle formation in the procedure using a natural extract.

### 2.2. Physicochemical Characterization of Nanoparticles

Transmission electron microscopy (FEI Tecnai G2 X-TWIN 200 kV microscope, FEI, Hillsboro, OR, USA) was used to characterize the nanoparticles’ size, size distribution, and morphology. For measurement, a drop of freshly prepared solution of nanoparticles was placed by casting on a carbon-covered 400-mesh Cu grid and left to air-dry. The aggregation and crystalline structures of the nanoparticles were measured by X-ray diffraction (XRD) (SuperNova Agilent Technologies diffractometer, Agilent, Santa Clara, CA, USA) with a Mo microfocused source (K_α2_ = 0.713067). Where a small quantity of nanoparticles was placed on a pin equipped with a nylon loop covered with highly viscous oil. Additionally, the hydrodynamic size of the nanoparticles was estimated by dynamic light scattering (DLS). Measurements were performed in water at room temperature (RT) by a NanoPlus spectrometer. UV–Vis spectroscopy of the Ag nanoparticles was also performed in a water medium as a solvent at RT (Able & Jasco V-670 spectrometer, Able & Jasco, Budapest, Hungary). Infrared (IR) spectra were collected in the 500–4000 cm^−1^ spectral range with a Nicolet 6700 spectrometer (ThermoScientific, Waltham, MA, USA) working in reflecting mode.

### 2.3. Antimicrobial Activity

The antimicrobial activity of the nanoparticles obtained in this study was determined as the minimum inhibitory concentration (MIC), following the Clinical and Laboratory Standard Institute (2021; https://www.clsi.org) protocols [34]. We tested the activity of the nanoparticles against human and animal pathogens representing Gram-postive (*Staphylococcus aureus* strain ATCC 6538, *Bacillus cereus* strain ATCC 10987) and Gram-negative (*Escherichia coli* strain ATCC 11229) bacteria, as well as fungus (*Candida krusei* strain ATCC 30135). The microorganisms were obtained from the American Type Culture Collection (ATCC). The microorganisms, stored at −80 °C in LB broth supplemented with glycerol (a ratio of 1:1), were inoculated onto nutrient agar (bacteria) or Sabouraud agar (fungus) and incubated overnight at 37 °C. The obtained nanoparticles were dissolved in dimethyl sulfoxide (DMSO) and serially diluted in Mueller–Hinton broth in the range of 1:4 to 1:2048 in 96-well microtiter plates, with final volumes of 100 μL. The overnight bacterial and fungal cultures in LB broth were suspended to a final optical density of 0.2–0.3 at 600 nm using a V-670 spectrophotometer (Jasco, Tokyo, Japan). They were then added at a volume of 100 μL to each well of a microtiter plate with the nanoparticles and incubated for 24 h at 37 °C. The turbidity of the microorganisms was observed visually after incubation. The MIC value is the highest nanoparticle dilution at which the growth of bacteria is not observed. All experiments were carried out in quadruplicate. Microorganisms cultured in Mueller–Hinton broth without nanoparticles were considered as a positive control. Microorganisms cultured in Mueller–Hinton broth supplemented with 10% DSMO were used as a solvent control. The microbiological media were supplied by Oxoid Ltd. (Basingstoke, UK).

## 3. Results and Discussion

### 3.1. Nanoparticle Characterization

#### Optical Characterization

**Figure 2:**
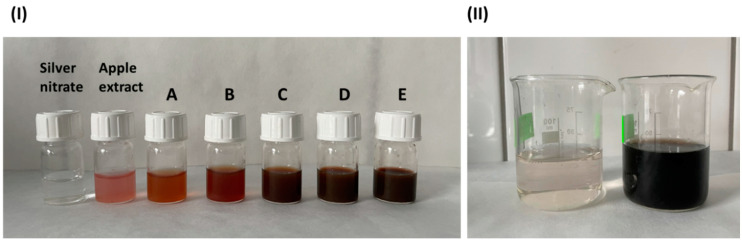
(**I**) Digital photo of vials with solutions containing (from left to right) silver nitrate solution, apple extract, and nanoparticles dispersed in the extract and mixed with AgNO_3_ at the following concentrations: (A) 0.25 mmol/L, (B) 0.5 mmol/L, (C) 1 mmol/L, (D) 2 mmol/L, (E) 4 mmol/L. (**II**) Digital photo of the two solutions before and after adding sodium hydroxide.

Figure 2I shows a digital photo of several vials containing solutions with nanoparticles reduced from precursors at appropriate concentrations that were dispersed in the apple extract. For comparison, the first vial contained a pure AgNO_3_ solution, while the second one consisted only of the apple extract. With a higher concentration of silver nitrate, the color changed from red to brown (vials A–E), proving the reduction of silver [33].

To check not only the precursor concentration but also the pH’s influence on the particles’ growth during the primary synthesis, described as solutions B and C, an appropriate amount of sodium hydroxide was added dropwise. As a consequence, an immediate reaction, signaled by a color change (Figure 2II), occurred, without the influence of the temperature, which indicated the reduction of silver to nanoparticle form [35].

### 3.2. Transmission Electron Microscopy (TEM)

**Figure 3:**
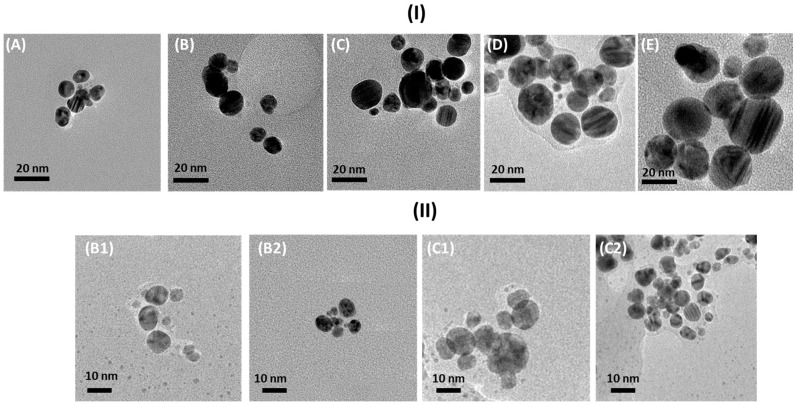
(**I**) TEM images with solutions containing apple extract and silver nitrate solutions with the following concentrations: (**A**) 0.25 mmol/L, (**B**) 0.5 mmol/L, (**C**) 1 mmol/L, (**D**) 2 mmol/L, (**E**) 4 mmol/L. (**II**) Synthesized nanoparticles in the presence of apple extract and sodium hydroxide: **(B1**) – 0.5 mmol/L (pH 6.2), (**B2**) 0.5 mmol/L (pH 9.6), (**C1**) 1 mmol/L (pH 6.2), (**C2**) 1 mmol/L (pH 9.6).

Details of the morphology and the size distribution of the synthesized nanoparticles were recorded by transmission electron microscopy (Figure 3). In row (I), a series displaying the precursor concentration dependence is present, whereas, in row (II), the pH influence is documented.

**Figure 4:**
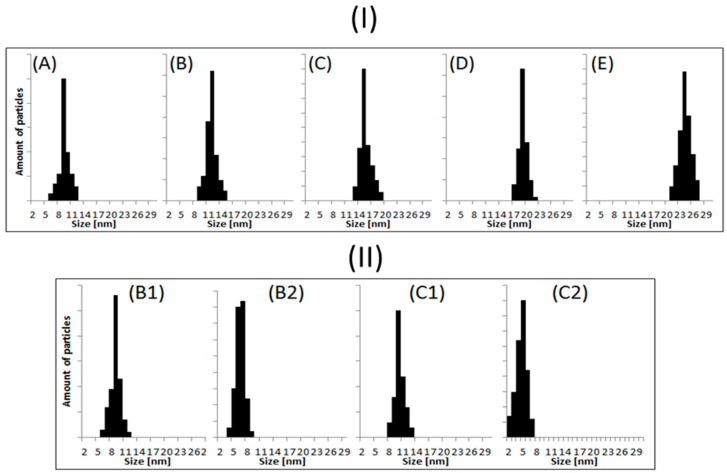
Size histograms determined on the basis of TEM images. (**I**) solutions containing apple extract and silver nitrate solutions with the following concentrations: (**A**) 0.25 mmol/L, (**B**) 0.5 mmol/L, (**C**) 1 mmol/L, (**D**) 2 mmol/L, (**E**) 4 mmol/L. (**II**) Synthesized nanoparticles in the presence of apple extract and sodium hydroxide: (**B1**)—0.5 mmol/L (pH 6.2), (**B2**) 0.5 mmol/L (pH 9.6), (**C1**) 1 mmol/L (pH 6.2), (**C2**) 1 mmol/L (pH 9.6).

The TEM images confirmed the presence of nanoparticles of various sizes, obtained by the green chemistry method using apple extract. The shape, size, and size distribution primarily depend on the concentration of the Ag precursor present in the primary mixture. Based on a qualitative and quantitative analysis of the TEM images, it was possible to calculate the diameters of the obtained nanostructures, determine the degree of agglomeration, and evaluate the size distribution of the obtained nano-objects. The nanoparticle size was estimated using the professional program *ImageJ 1.54d* [33]. The shapes and morphologies of the nanoparticles with an added NaOH solution were also imaged to check the pH effect. The numerical values obtained for each solution are summarized in Table 2. The evaluated histograms, based on the TEM images depicted in Figure 3, are presented in a respective series in Figure 4.

The plotted histograms (Figure 4I) confirm quantitatively that, with the growth of the silver core precursor concentration, the average size of the nanoparticles increased. The measurements were conducted on more than 100 particles to ensure statistical reliability and the accurate representation of the data in the histograms. The depicted histograms (Figure 4II) show, on the other hand, that a change in pH affects the particle quality as well. In both series, with an increase in the pH value, the particle size decreased [9,33]. Therefore, the combination of the concentration and pH allows the growth of particles of the required size.

Summarizing the obtained TEM images leads to the conclusion that the smallest nanoparticles were formed in the sample at a concentration of 0.25 mmol/L (9 nm ± 1 nm). In comparison, the largest ones were formed at a concentration of 4 mmol/L (22 nm ± 1 nm). This proves that the lower the concentration of the ion precursor solution, the smaller the nanoparticles formed [36]. As can be seen in the images (Figure 4), the synthesized nanoparticles were not in direct contact with each other. Therefore, they did not form tight aggregates, which may have been caused by the efficient surface stabilization by the compounds present in the extracts. In some TEM images, surface layers around the particles are clearly visible. This quantitatively proves the nanoparticles’ stabilization by the compounds present in the apple extract [37]. It is also seen that, with increasing amounts of the inorganic core precursor (silver nitrate) present in the reaction mixture, the average size of the particle rises. Such observations suggest that the growth of existing centers in the composition is more favorable than creating new seeds [38,39].

Other studied modifications of the synthesis process show that the more alkaline the environment, the smaller the particles produced. The shape of the obtained nanoparticles in the procedure with NaOH is more homogeneous compared to that seen in other trials [40]. After adding the sodium base, there is a correlation in the particles’ growth, meaning that smaller particles are formed at a lower core precursor concentration and in a more alkaline environment [41].

### 3.3. X-Ray Diffraction (XRD)

**Figure 5:**
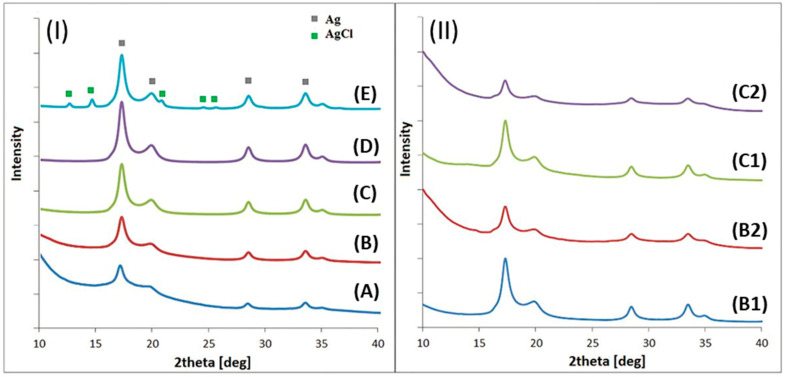
Diffractograms of nanoparticles synthesized in the presence of apple extract and different amounts of Ag precursor (**I**) and different pH values (**II**).

X-ray diffraction was used to identify the crystal structures presented in the system and estimate the synthetized nanoparticles’ sizes. The data obtained for all samples from both diffractogram series are collected in Figure 5. This confirms that the synthesized nanoparticles were in the form of nanocrystals of various sizes and compositions (metallic Ag or AgCl) [33].

The XRD data obtained for the nanoparticles of both series (where either the ratio of the precursor particles to the surfactant (Figure 5I) or the pH (Figure 5II) was varied) show that diffraction peaks were obtained from two crystallographic systems: metallic silver and silver chloride. The metallic Ag diffraction peaks were on the 2 theta axis at positions 17.27°, 19.97°, 28.39°, and 33.42°, which correspond to the planes hkl according to Miller’s nomenclature: (111), (200), (220), and (311), respectively [33,42]. The silver chloride diffraction peaks were identified at the angle values of 12.83°, 14.53°, 21.02°, 24.71°, 25.83°, and 33.56°, which correspond to the following hkl planes: (111), (200), (220), (311), (222), and (420), respectively [41,42]. The relevant PDF card numbers for the identified phases are as follows: for metallic silver (Ag), ICDD PDF Card No. 04-0783; for silver chloride (AgCl), ICDD PDF Card No. 31-1238 [43,44].

The qualitative composition of the diffractograms from frame (I) allows us to conclude that an increase in the amount of the Ag precursor causes the shape of the peaks to change. With the crystallinity improvement, the pattern intensity is much higher, and the peaks become narrower, which directly indicates particle growth or better crystallinity. Additionally, the AgCl patterns’ traces were present only for the case of synthesis with the largest amount of AgNO_3_ [45]. From frame (II), it is clear that the more acidic the solution, the larger the particles grown [41] and the better the crystallinity of the obtained particles.

The average size of the nanoparticles synthesized using apple extract, along with the lattice parameter and phase fraction, was determined by matching the diffraction data with a theoretical model. The application of the modified Scherrer formula–Williamson–Hall (1) equation provided quantitative results, which are summarized in Table 3 [46].

(1) $$\beta \cos\theta =\left(\frac{0.9\lambda }{D}\right)+\left(4\varepsilon \sin\theta \right)$$

where

*D*—grain size [Å];
λ—wavelength (for Mo source, this is 0.7136 Å);
*β*—full width at half maximum intensity of the peak [rad];
*ε*—strain;
*Ɵ*—diffraction angle [rad].

In our study, the lattice parameters were calculated by refining the X-ray diffraction (XRD) data using PCW (PowderCell for Windows). The quantitative phase analysis was conducted by fitting the experimental diffraction patterns to reference patterns available in crystallographic databases, ensuring the accurate determination of the phase fractions. Regarding the crystallite size evaluation, we applied the Williamson–Hall equation to provide a comprehensive analysis [47]. Broadly using the Scherrer equation allows us only to estimate the crystallite size based on one peak’s broadening due to the crystallite size effect, while the Williamson–Hall method employs both size-induced and strain-induced broadening [48]. This approach allowed us to assess the influence of strain on the synthesized nanoparticles.

The structure of metallic silver has regular symmetry, centered on the walls (a = 0.409 nm [41,42,44]). The experimentally determined lattice parameter ranges from 4.062 to 4.092 ± 0.002 Å. The structure of the silver chloride lattice has also regular symmetry, and the experimentally determined lattice parameter is equal to 5.490 ± 0.002, compared to the literature value of 5.549 Å [31]. Both phases’ growth is in agreement with theoretical data [49,50]. Plant extracts contain various components that can serve as a source of chloride ions in the reaction mixture, facilitating the formation of silver chloride nanoparticles [51,52].

The XRD crystallite size measurements confirm also that the smaller the Ag ion precursor concentration used, the smaller the crystallites obtained. The smallest nanoparticles were grown at a concentration of 0.25 mmol/L (8 nm ± 1), showing, however, a metallic silver structure. For samples B1 and C1, both with a pH value of 6.2, sharper peaks are noticeable (Figure 5II), indicating better crystallinity or a larger particle size with a metallic silver structure. The signals are not very sharp for samples B2 and C2, which have more alkaline pH values equal to 9.6. This suggests the lower efficacy of silver nanoparticle formation (see Figure 5II).

The diffraction measurements show that the reaction environment during synthesis is essential in crystallite formation (Table 3). The value determined using XRD data confirms that the more alkaline the nanoparticle formation medium and the lower the concentration of the Ag ion precursor used, the smaller the nano-objects were formed [47]. Smaller nanoparticles were obtained in the alkaline environment at pH 9.6 than in the more acidic environment (6.2). All conclusions obtained based on X-ray diffraction are in line with the TEM results.

### 3.4. Dynamic Light Scattering (DLS)

**Figure 6:**
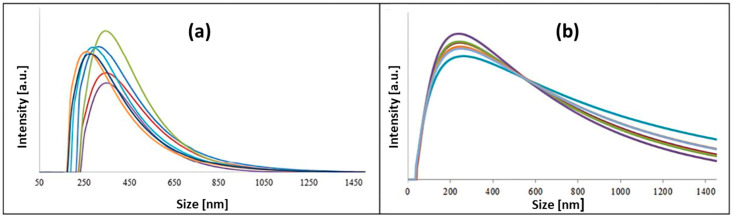
(**a**) Examples of DLS curves consisting of a cascade of seven measurements of the synthesized nanoparticle solution (sample B) in the presence of apple extract. (**b**) Examples of DLS curves of the synthesized nanoparticle solution with NaOH (sample B1) in the presence of apple extract.

The DLS technique is used in a given nanoparticle solution to determine the average hydrodynamic size, the size distribution, and the tendency for aggregate formation [53]. The DLS experimental data (Figure 6) confirm that the nanoparticles are surrounded by active compounds in the plant extracts, as seen in the TEM and XRD data. However, narrow peaks were noted for the nanoparticle solutions obtained in the presence of apple extract, which proves the small size distribution of the obtained nanostructures [54].

The stability of the nanoparticle–plant extract system is also confirmed by the measurements performed in the series. For this purpose, seven scans of each sample were recorded, but only one example is presented (Figure 6a). In subsequent measurements, the average size of the created objects slightly increases, which may suggest an agglomeration process [55]. In addition, the stability of the nanoparticles in the solution decreases, and the central peak becomes blurred [54,56].

To correctly interpret the results of DLS analyses, an appropriate method for their presentation should be used. Inappropriate processing may cause the presence of a small volume percentage of larger nanoparticles to obscure the number of smaller nanoparticles completely. The size dispersion of the identified objects in individual samples is related to the hydrodynamic radius, i.e., not only the size of the inorganic core but also the compounds acting as surfactants and stabilizers, as well as other layers formed around the surface [53]. The size, estimated by simulation, is defined for a sphere model with the same diffusion coefficient as the measured nanostructure. These factors may cause the nanoparticle diameter to differ significantly from those determined by TEM [33].

The corresponding curves of the biosynthesized nanoparticles in the presence of a suitable amount of NaOH were recorded and are shown in Figure 6b. The particle size distribution obtained in DLS measurements is strongly asymmetric towards larger objects and does not accurately reflect the correct number of particles of a given size. This is because the particle size matching models in DLS assume that all particles involved in the experiment are only spherical. Experimental data recorded with DLS confirm that the nanoparticles are surrounded by active compounds present in plant extracts [53]. The maximum position is stable, which may indicate a homogeneous size among the nano-objects [57].

### 3.5. UV–Vis Spectroscopy

The typical maximum absorption in the UV–Vis spectrum of pure silver nanoparticles is around 420 nm due to plasma edges present in this region [27,33,58]. The registration of such a signal indicates that the particles are well dispersed and do not aggregate [33]. Small-sized nano-objects generate peaks in the vicinity of 400–420 nm. When larger forms are present, as in sample E by TEM and XRD, this particles are characterized by a spectrum with the maximum shift towards the longest wavelengths (red shift). This confirms the observation that, as the particles grow, the peak position corresponding to the absorption maximum shifts to longer wavelengths [59].

**Figure 7:**
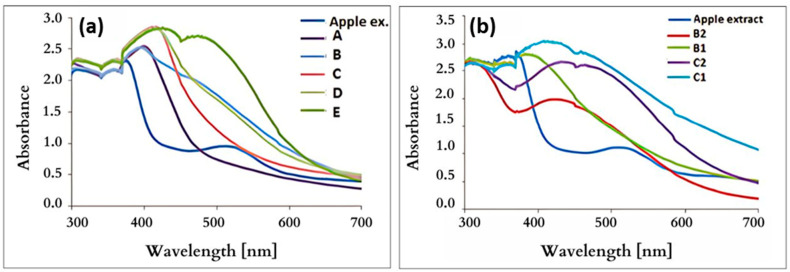
UV–Vis spectra of nanoparticles synthesized in the presence of (**a**) apple extract, (**b**) apple extract and NaOH.

The absorption maxima were measured at around 420 nm (as is seen in Figure 7), confirming the synthesis of the desired nanoparticles [27,33]. Moreover, at a concentration of 4 mmol/L, an additional band is visible at around 500 nm. This may indicate the presence of silver chloride, as its crystalline form was noticed in the XRD measurements [56].

The shift in the peak maximum towards higher wavelengths indicates the nanoparticles’ growth [33,38]. The positions of the maximum peak in the UV–Vis curves recorded for the nanoparticles obtained using apple extract were identified in the range of about 410–425 nm (Table 4). The positioning and shape of the maxima depend on both the size and effective shape of the nanostructures [38].

There is also a visible dependence on the increasing pH in the given UV–Vis spectra. The more alkaline the pH is, the higher the absorption maximum, which may indicate a smaller percentage of produced silver nanoparticles. For samples B1 and C1, which have an acidic pH (6.2), the maximum absorption is in the range for silver nanostructures (about 420 nm) [54]. Hence, the alkaline environment influences the wavenumber shift in UV–Vis spectroscopy. An increase in pH can lead to an increase in the nucleation center, thereby increasing the reduction of the metal ion to nanoparticles [60,61]. After the TEM and XRD measurements, it can be concluded that the smaller the size, the more noticeable the shift towards shorter wavelengths. It can also be stated that the maximum absorption rises compared with an increasing pH [41].

### 3.6. Infrared Spectroscopy

**Figure 8:**
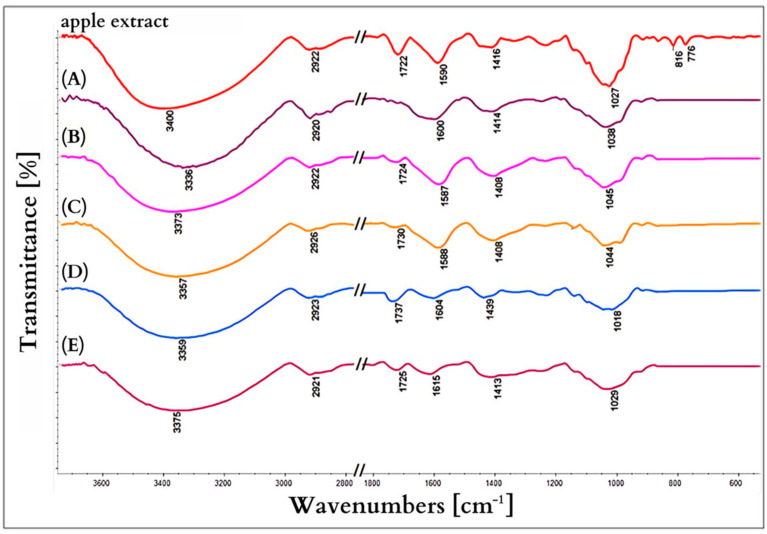
IR spectra of synthesized nanoparticles A–E.

Figure 8 shows the IR spectra of the nanoparticles to compare the signal positions. The wide band at 3330 cm^−1^ corresponds to O-H bonds from water particles [62]. The weak peaks at 2924 cm^−1^ for aerobic conditions may show vibrations of the aliphatic C-H group [63]. The vibration at about 1600 cm^−1^ can be attributed to the N-H stretching vibration in the amide bonds of the proteins (present in the plant extract), which plays a role in the stability/confinement of the silver nanoparticles [63]. The registered bands from 1700 to 1500 cm^−1^ result from various types of double bond stretching vibrations (C=O, C=C, C=N). All these signals originate from the apple extract [64].

The vibration bands corresponding to O–H, C–H bonds, the C=C ring, C–OH, and C–C come from water-soluble compounds such as ascorbic acid, flavonoids, and polyphenols present in apple [64]. These results are consistent with studies that have shown high content of ascorbic acid and flavonoids in apple fruits [65,66]. It can therefore be concluded that these water-soluble polar compounds are responsible for both the reduction of Ag(I) and the effective stabilization or capping of the prepared silver nanoparticles.

### 3.7. Antimicrobial Activity of Silver Nanoparticles Obtained with Apple Extracts

The selection of *B. cereus* ATCC 10987, *S. aureus* ATCC 6538, *E. coli* ATCC 11229, and *C. krusei* ATCC 30135 was based on their significance in terms of pathogenicity and resistance to antimicrobial agents. For instance, skin infections are often associated with *S. aureus* and *C. krusei.*, and alimentary tract infections are caused by *B. cereus* and *E. coli*, while food poisoning is linked to *S. aureus* and *B. cereus* toxins. Thus, the inclusion of these four strains allowed for a comprehensive assessment of the antimicrobial properties of the synthesized silver nanoparticles. Each of them could have exhibited different behavior, as the set included two Gram-positive bacteria, one Gram-negative bacterium, and one opportunistic yeast. This diverse selection provided a broad spectrum of microorganisms, enabling a more thorough evaluation of the potential antiseptic and therapeutic applications.

In our study, we focused on determining the minimum inhibitory concentration (MIC), as this method allows for a more precise assessment of the antimicrobial activity compared to diffusion-based methods, such as the disk diffusion or well diffusion methods. The MIC determines the exact concentration of an antimicrobial substance at which microbial growth is inhibited (which was our objective) [66]. In the disk diffusion method, only the zone of inhibition around the disk is assessed, making it a qualitative rather than quantitative method. The MIC is a more precise, reproducible, and quantitative approach, whereas disk diffusion is more commonly used as an initial screening test [67].

The Ag nanoparticles obtained in this study with the use of apple extracts as reducers demonstrated activity against the bacteria and fungus (Table 5). The nanoparticles were the most active against the Gram-positive bacterium *B. cereus*, especially concerning specimens C, D, and E, for which the inhibitory activity was noted at the nanoparticle dilutions of 1:512 and 1:1025. For *S. aureus*, the nanoparticles demonstrated lower inhibitory activity. These Gram-positive cocci did not grow in the nanoparticle dilutions varying from 1:32 to 1:256. The lowest activity of the obtained particles was noted against Gram-negative bacteria, i.e., *E. coli*. The inhibitory effect was observed when the nanoparticles were diluted from 1:4 (sample E) to 1:64 (sample C). In contrast, the *C. krusei* cells were more sensitive to the nanoparticles, since the dilutions with no growth varied from 1:32 to 1:512.

We also determined whether the tested microorganisms were affected by the nanoparticles or the plant extract itself. In our study, we did not observe any antimicrobial activity of the apple extract itself. Thus, for the given samples, the interaction of the microorganisms with the plant matrix itself (without the presence of nanoparticles) was negligible. Based on this, we can conclude that nanoparticles synthesized by adding apple extract showed antibacterial activity, but not the extract itself. The MIC method was also used to evaluate the silver nitrate solution to assess its antimicrobial activity and compare it with that of silver nanoparticles. AgNO_3_ exhibited negligible antimicrobial activity against *B. cereus*, *S. aureus*, and *C. krusei*, but it did not inhibit the growth of *E. coli*, unlike the silver nanoparticles at various concentrations. These results indicate that the mere presence of Ag^+^ ions may have some antimicrobial effect. However, the effect is significantly stronger in the case of nanoparticles. This is because the smaller the nanoparticle size, the greater the release of Ag^+^ ions, leading to more effective microbial deactivation [68,69].

It should be remembered that ionic silver is released from nanoparticles when dissolved in water or when they penetrate cells [70]. This results in the nanoparticles having higher antibacterial activity than free silver ions. For this reason, antibacterial properties are attributed to physical characteristics [71]. The electrostatic forces that arise between nanoparticles with a positive zeta potential when encountering bacteria with a negative surface charge promote attraction, causing closer interactions between the two objects and, consequently, the easier penetration of bacterial membranes [72]. An essential aspect of the antimicrobial potential of nanoparticles may be the correlation between the inhibitory effect and the dilution of the silver nanoparticle solution. However, the species of bacteria plays an important role here as well. Studies on the antimicrobial activity of silver nanoparticles against *B. cereus* and *E. coli* showed that *B. cereus* was inhibited in the presence of low concentrations of silver (high dilution). At the same time, the reduction in *E. coli* growth was less noticeable. Bacterial cell growth decreased dramatically when the concentration of silver nanoparticles increased (lower dilution). Similar observations were also noted for *C. krusei*, for which the higher dilution of nanoparticles in the sample led to stronger antifungal potential.

There was a noticeable correlation as sample E, which had the largest size, exhibited the highest antimicrobial activity. In sample E, the presence of the AgCl phase was also detected, which may have contributed to the enhanced antimicrobial activity. It is worth noting that AgCl (silver chloride) releases Ag^+^ ions more easily than metallic silver (Ag^0^), which accounts for its stronger antibacterial properties. In the structure of metallic silver, the atoms are connected by strong metallic bonds in a crystalline lattice, whereas, in AgCl, Ag^+^ and Cl^−^ ions are held together by electrostatic interactions (ionic bonds). Metallic silver requires oxidation to Ag^+^, which does not occur spontaneously, whereas AgCl can partially dissolve and release Ag^+^ into the solution [73].

However, the primary factor influencing this activity is the size of the obtained nanoparticles. Our other studies focused on different extracts with varying AgCl content, and the conclusion was that the smaller the nanoparticles, the stronger the antimicrobial activity [33]. A study of the effects of silver nanoparticles of various shapes on *E. coli* and *B. cereus* showed that silver nanoparticles undergo interactions that depend on their size and concentration/dilution. The higher the concentration, the stronger the biocidal effect compared to smaller nanoparticles [74]. It has been proven that silver nanoparticles are an effective antibiotic against pathogens. In this case, the concentration of silver nanostructures is more critical for its effectiveness than its size [75].

## 4. Conclusions

Green chemistry is an environmentally friendly method of producing materials that reduces both the consumption and production of hazardous substances from biodegradable substrates [76]. The silver nanoparticles synthesized in this study using apple extract were homogeneous and did not tend to agglomerate. The characteristics of the obtained nanoparticles showed that the lower the concentration of the silver ion precursor used, the smaller the nanoparticles produced. Moreover, the diffraction measurements showed that the reaction environment during synthesis is vital in the formation of crystallites. The influence of the pH on the reaction environment is also crucial in forming nano-objects. The more alkaline the environment was, the smaller the nano-objects formed, while the more acidic the environment was, the larger the crystallites observed. The UV–Vis results proved that nano-objects of a small size generate peaks in the vicinity of 410–425 nm. At the same time, larger forms are characterized by a spectrum with a maximum shifted towards longer wavelengths. Data recorded by DLS confirmed that the nanoparticles were surrounded by active compounds in the apple extract. The nanoparticles with apple extracts as reducers demonstrated higher activity against Gram-positive bacteria and fungus than against Gram-negative bacteria. The development of nanotechnology in green chemistry processes will gradually increase with technological advances, being a key aspect in developing new synthesis processes for nano-objects [77].

## Figures and Tables

**Table 1:** Assessment of the samples according the resulting pH of the solutions.

Sample	Concentration of AgNO_3_ [mmol/L]	Amount of NaOH [mL]	pH of the Solution
B1	0.5	3	6.2
B2	0.5	6	9.6
C1	1	3	6.2
C2	1	6	9.6

**Table 2:** Estimated size and shape of nanoparticles based on TEM images.

Sample	Size ± 2 nm	Shape
A	9	Spherical
B	12	Spherical
B1	9	Ellipsoidal
B2	7	Ellipsoidal or spherical
C	15	Spherical
C1	11	Ellipsoidal
C2	6	Spherical
D	19	Ellipsoidal or spherical
E	24	Ellipsoidal or spherical

**Table 3:** The nanoparticles’ crystallite size, phase percentage, and lattice parameters calculated from XRD data.

Sample	Size ± 2 [nm]	Lattice Parameter Ag ± 0.002 [Å]	% Ag /%AgCl ± 1%
A	8	4.091	100
B	13	4.092	100
B1	10	4.072	100
B2	7	4.091	100
C	16	4.090	100
C1	12	4.072	100
C2	8	4.090	100
D	19	4.091	100
E	20	4.062 /5.490	89 /11%

**Table 4:** Position of the maximum absorption peak of silver nanoparticles from UV–Vis spectroscopy.

Sample	A	B	B1	B2	C	C1	C2	D	E
Maximum absorption [nm]	411	412	400	417	419	420	428	420	425

**Table 5:** The antimicrobial potential demonstrated as the minimal inhibitory concentration (MIC), presented as the dilution of nanoparticles synthesized with apple extract and the extract itself.

Microorganism	Specimen						
	Extract Apple	AgNO_3_	Sample A	Sample B	Sample C	Sample D	Sample E
*B. cereus* ATCC 10987	-	1:128	1:128	1:128	1:512	1:512	1:1024
*S. aureus* ATCC 6538	-	1:32	1:128	1:32	1:64	1:256	1:256
*E. coli* ATCC 11229	-	-	1:8	1:32	1:64	1:8	1:4
*C. krusei* ATCC 30135	-	1:32	1:32	1:64	1:256	1:512	1:512

## Data Availability

The data sets generated during the current study are available from the corresponding author on reasonable request.
